# Draft genome data of *Ochrobactrum anthropi* strain Nas42 from estuarine sediments of the Red River Delta, Vietnam

**DOI:** 10.1016/j.dib.2026.112689

**Published:** 2026-03-14

**Authors:** Duong Huy Nguyen, Nathalie Pradel, Sandrine Chifflet, Marc Tedetti, Ngoc Bich Pham, Van Ngoc Bui

**Affiliations:** a Institute of Biology (IB), Vietnam Academy of Science and Technology (VAST), Hanoi, 100000, Vietnam; b Aix-Marseille Univ, Université de Toulon, CNRS, IRD, MIO UM 110, 13288, Marseille, France; c Chemical and Environmental Technology (CET) Department, University of Science and Technology, of Hanoi (USTH), Vietnam Academy of Science and Technology (VAST), Hanoi, 100000, Vietnam; d Graduate University of Science and Technology (GUST), Vietnam Academy of Science and Technology (VAST), Hanoi, 100000, Vietnam

**Keywords:** Ochrobactrum, Metals, Whole-genome sequencing, Red River Delta, Illumina NovaSeq

## Abstract

This dataset presents the draft genome of *Ochrobactrum anthropi* strain Nas42, isolated from metal-contaminated estuarine sediments of the Red River Delta, Vietnam. Genomic DNA was extracted from the isolate and sequenced using the Illumina MiSeq platform, followed by de novo assembly and functional annotation. The generated dataset comprises a 4605,092 bp draft genome assembled into 27 contigs with a GC content of 56.11%, containing 4356 protein-coding sequences. Notably, *in silico* whole-genome comparison supported its taxonomic assignment to *O. anthropi*, demonstrating 99.37% Average Nucleotide Identity (ANI) with strain PBO and the absence of IS711 insertion sequences, a genomic feature characteristic of *Brucella* species but absent in *Ochrobactrum*. Additionally, the annotated dataset highlights 45 genes associated with metal resistance, including those encoding resistance to cobalt, zinc, cadmium, and copper. This genomic resource can be reused by the scientific community to investigate metal resistance determinants, explore microbial adaptation mechanisms in polluted estuarine environments, and support phylogenomic analyses of the *Ochrobactrum–Brucella* complex.

Specifications Table

**Table:** 

Subject	Biology
Specific subject area	Bacterial genomics
Type of data	Table, Figure, Raw, Analyzed, Processed and Deposited
Data collection	Genomic DNA of *Ochrobactrum anthropi* strain Nas42 was extracted from a metal-selective culture of sediments from the Red River Delta (Vietnam), quality-checked by spectrophotometry and agarose gel electrophoresis, and prepared for whole-genome sequencing using the NEBNext dsDNA Fragmentase and NEBNext Ultra II DNA Library Prep Kit for Illumina (NEB, USA). Sequencing was performed on the Illumina MiSeq platform. The raw reads were trimmed using Trimmomatic v0.39, assembled with SPAdes v3.2.0 and Unicycler v0.5.1, and subjected to genome quality assessment with QUAST v5.3.0, BUSCO v5.8.0, and CheckM2 v1.0.2. Functional annotation was carried out using Prokka v1.14.6, COG, KEGG, GO, and RAST, followed by whole-genome-based taxonomic analysis using TYGS, ANIb, and IS711 screening.
Data source location	Institute of Biology, Vietnam Academy of Science and Technology, Hanoi, Vietnam
Data accessibility	Repository name: Data identification number: BioProject PRJNA1372985, with BioSample SAMN53633865. Direct URL to data: https://www.ncbi.nlm.nih.gov/sra/?term=SAMN53633865
Related research article	None

## 1 Value of the Data

• These data provide the whole draft genome sequence of *Ochrobactrum anthropi* strain Nas42 isolated from sediments of the Red River Delta, Vietnam, which will be valuable for molecular taxonomy and genome-based phylogenetics of environmental *Ochrobactrum* and related genera.
• New insights for distinguishing *Ochrobactrum* and *Brucella* genera.
• The genomic dataset enables researchers to investigate and compare metal resistance determinants and stress-adaptation pathways in *O. anthropi* and other bacteria from polluted ecosystems.
• The high-quality genome and curated annotations offer important insights for understanding the mechanisms underpinning metal tolerance and for exploring the potential of O. anthropi strain Nas42 in bioremediation applications.

## 2 Background

*Ochrobactrum anthropi* is a Gram-negative, non-fermenting bacterium phylogenetically related to the genus *Brucella* but distinguished by its remarkable ecological versatility, inhabiting diverse niches such as soil, water, and sediments [1,2]. In Vietnam, the Red River Delta faces severe anthropogenic pressure from mining, metallurgical processing, and agriculture, leading to the accumulation of toxic metals, particularly arsenic and cadmium, in aquatic and sedimentary environments [3,4].

Microbial communities persisting under such chronic contamination often evolve distinct genomic adaptations that facilitate survival under metal stress [5,6]. While *O. anthropi* is phylogenetically close to *Brucella*, recent analyses support maintaining their taxonomic distinction based on key genomic features, including larger genome size and the absence of the IS711 insertion sequence [7]. Despite the increasing recognition of *O. anthropi* as an opportunistic pathogen in clinical settings, genomic data on environmental isolates from Asian estuaries, particularly in Vietnam, remain limited. Consequently, the genome sequence of strain Nas42 provides a critical resource for elucidating the genetic determinants of contaminant tolerance and reinforcing the taxonomic placement of *O. anthropi* within this impacted ecosystem.

## 3 Data Description

**Fig. 1:**
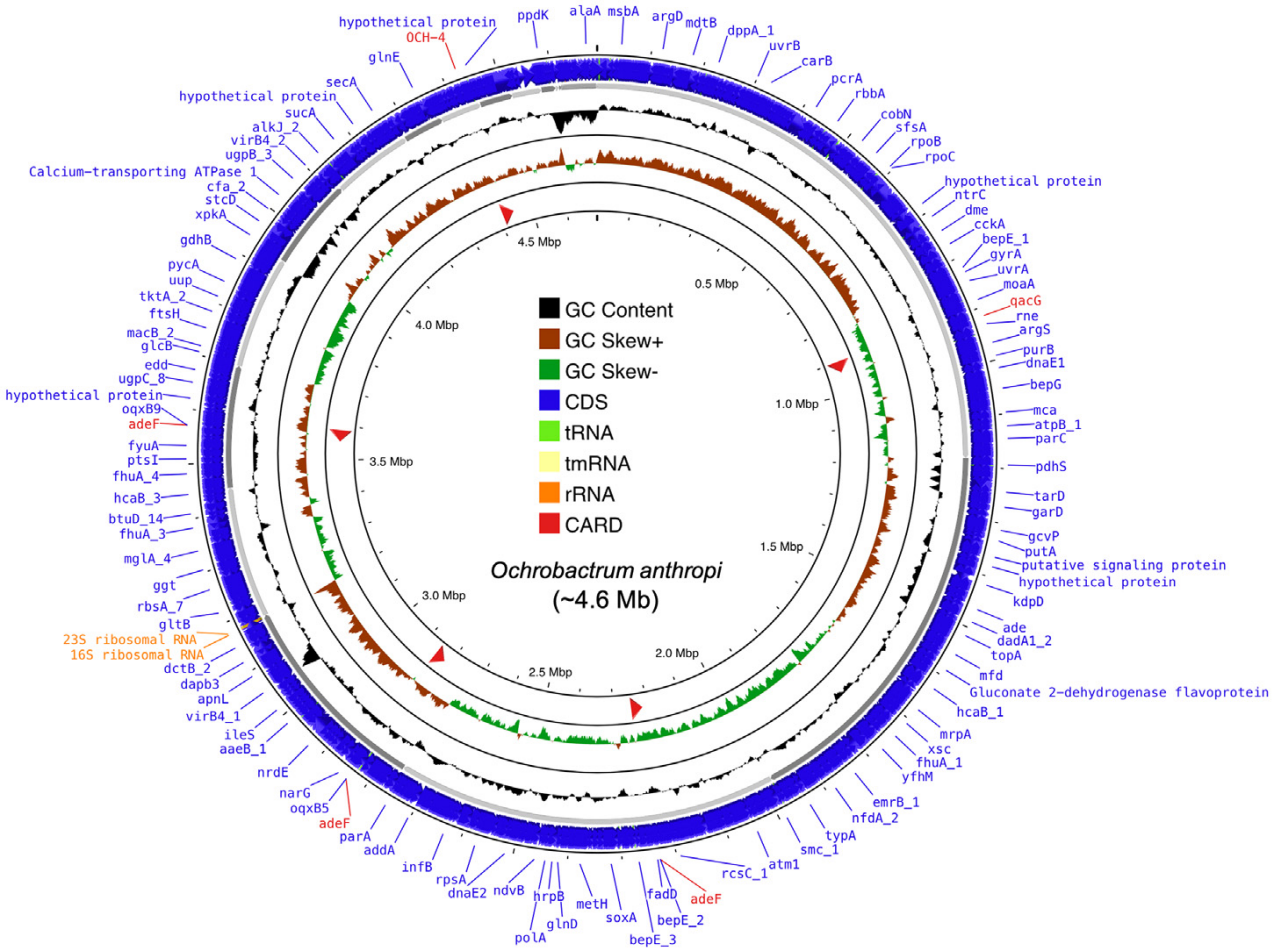
Circular genome map of *Ochrobactrum anthropi* strain Nas42 constructed using the CGView server (https://proksee.ca/, accessed 20 November 2025). CDSs are represented by blue arrows, while contigs are represented by grey arrows. Brown peaks represent GC skew+, green peaks represent GC skew-, and black peaks represent GC content.

This article provides whole-genome sequencing information for *Ochrobactrum anthropi* strain Nas42, isolated from sediments of the Red River Delta, Vietnam. The draft genome (Fig. 1) was generated using the Illumina MiSeq platform and assembled into 27 contigs with a total length of 4605,092 bp and a GC content of 56.11 %. The genomic features and functional annotations are summarized in Table 1.

**Fig. 2:**
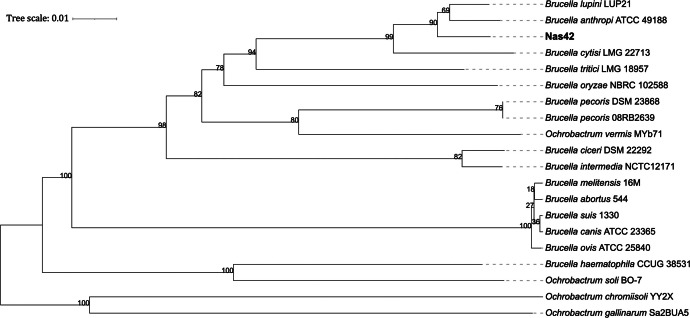
Phylogenomic tree constructed using whole-genome sequence data from strain Nas42 and closely related type strains on the TYGS platform. Branch numbers were determined based on pseudo-bootstrap support values greater than 18 % from 1000 replicates using Genome Blast Distance Phylogeny (GBDP), with average branch support at 78.2 %.

**Table 1:** Summary of genomic features and assembly statistics for strain Nas42.

Genome assembly features	Values
Total assembly size (bp)	4605,092
Total assembly size (≥ 1000 bp)	4602,169
Total assembly size (≥ 50,000 bp)	4535,153
Number of contigs (≥ 1000 bp)	21
Number of contigs (≥ 50,000 bp)	13
Total number of contigs	27
Largest contig (bp)	1181,188
GC-content (%)	56.11
N50 contig length (bp)	775,761
L50 contig count	3
Total length (bp)	4604,168
Genome coverage	206.56x
***Completeness evaluation (%)***	
Completeness	99.1
Complete and single-copy BUSCOs	98.2
Complete and duplicated BUSCOs	0.9
Fragmented BUSCOs	0.9
Missing BUSCOs	0.0
Completeness (CheckM)	99.14
Contamination (CheckM)	0.86
***Repeat annotation***	
Microsatellites (Number of elements)	1
Minisatellites (Number of elements)	48
Total tandem repeat sequence (%)	0.043
Total repeat length (bp)	2001
***Genomic annotation***	
Genes (total)	4460
CDSs (total)	4356
tRNA	50
rRNA (5S, 16S, 23S)	3 (1, 1, 1)
tmRNA	1

The phylogenomic tree of strain Nas42 and its closest relatives (Fig. 2) shows that Nas42 clusters with “*Brucella anthropi*” ATCC 49,188 and *Brucella lupini* LUP21 with 90 % bootstrap support.

The CheckM and BUSCO analyses are presented in Table 1, where the genomic features are summarised.

Comparative genomic metrics between strain Nas42 and reference genomes, including IS711 sequence screening and pairwise ANI values, are summarized in Table 2.

**Table 2:** Summary of comparative analysis and identification of the strain Nas42 genome.

Genome	IS711 sequence	BLAST			Prediction	% ANI (compared with Nas42)	Genome size
		Hits	% Identity	Coverage			
**Strain Nas42**	NO	0	NA	NA	*Ochrobactrum*	100	4.69
*Brucella anthropi* PBO	NO	0	NA	NA	*Ochrobactrum*	99.37	4.86
*Brucella anthropi* CGMCC 1.17299	NO	0	NA	NA	*Ochrobactrum*	98.18	5.23
*Brucella lupini* LUP21	NO	0	NA	NA	*Ochrobactrum*	98.00	5.61
*Brucella anthropi* FDAARGOS 1039	NO	0	NA	NA	*Ochrobactrum*	97.56	5.17
*Brucella anthropi* ATCC 49,188	NO	0	NA	NA	*Ochrobactrum*	97.50	5.21
*Ochrobactrum vermis* MYb71	NO	0	NA	NA	*Ochrobactrum*	88.43	5.40
*Ochrobactrum soli* BO-7	NO	0	NA	NA	*Ochrobactrum*	83.23	5.02
*Brucella canis* ATCC 23,365	YES	6	99.052	100	*True Brucella*	82.79	3.31
*Brucella melitensis bv.*1str.16M	YES	7	98.695	100	*True Brucella*	82.78	3.29
*Brucella suis* 1330	YES	7	99.05	100	*True Brucella*	82.72	3.32
*Brucella ovis* ATCC 25,840	YES	40	100	100	*True Brucella*	82.66	3.28
*Ochrobactrum* sp. BTU1	NO	0	NA	NA	*Ochrobactrum*	81.14	5.88
*Ochrobactrum chromiisoli* YY2X	NO	0	NA	NA	*Ochrobactrum*	80.71	4.65

**Fig. 3:**
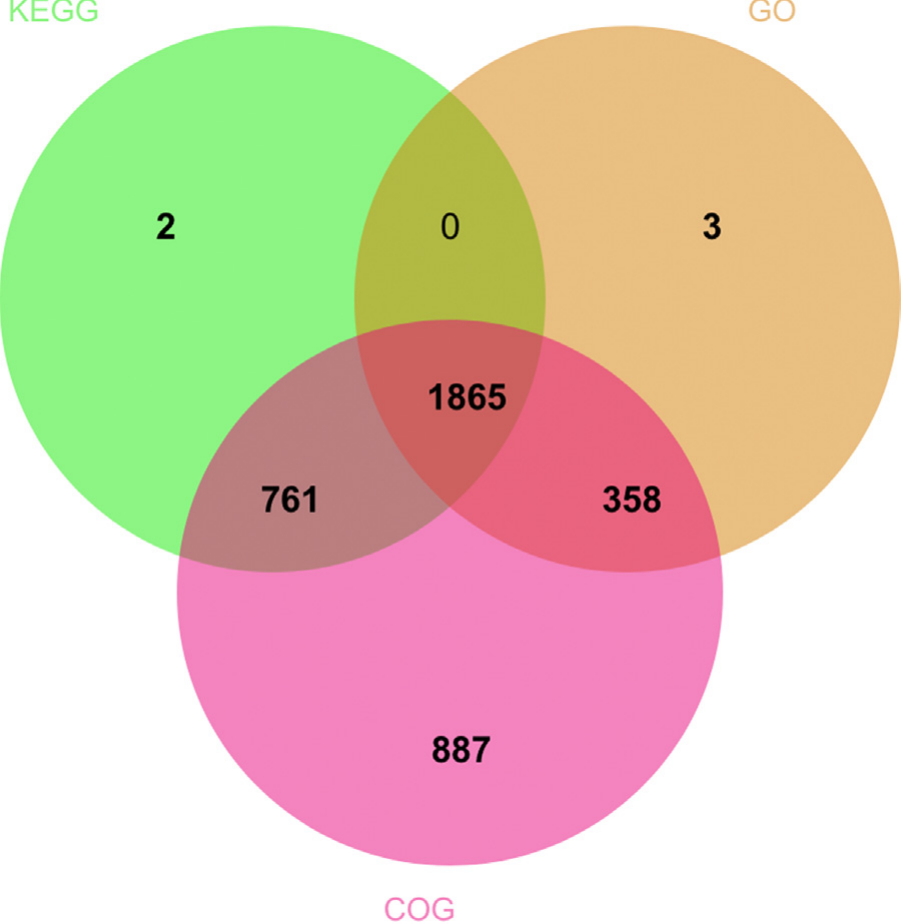
Venn diagram showing the number of shared and unique gene annotations across the KEGG, GO, and COG databases.

Functional annotation of the strain Nas42 genome using eggNOG-based pipelines assigned the majority of predicted CDSs (Coding DNA sequences) to at least one of the COG (Clusters of Orthologous Genes), KEGG pathway, or GO (Gene Ontology) databases. In total, 3876 genes (approximately 89 % of all CDSs) were annotated in at least one database, and 1865 genes were shared across all three resources, as summarized by the Venn diagram (Fig. 3).

**Fig. 4:**
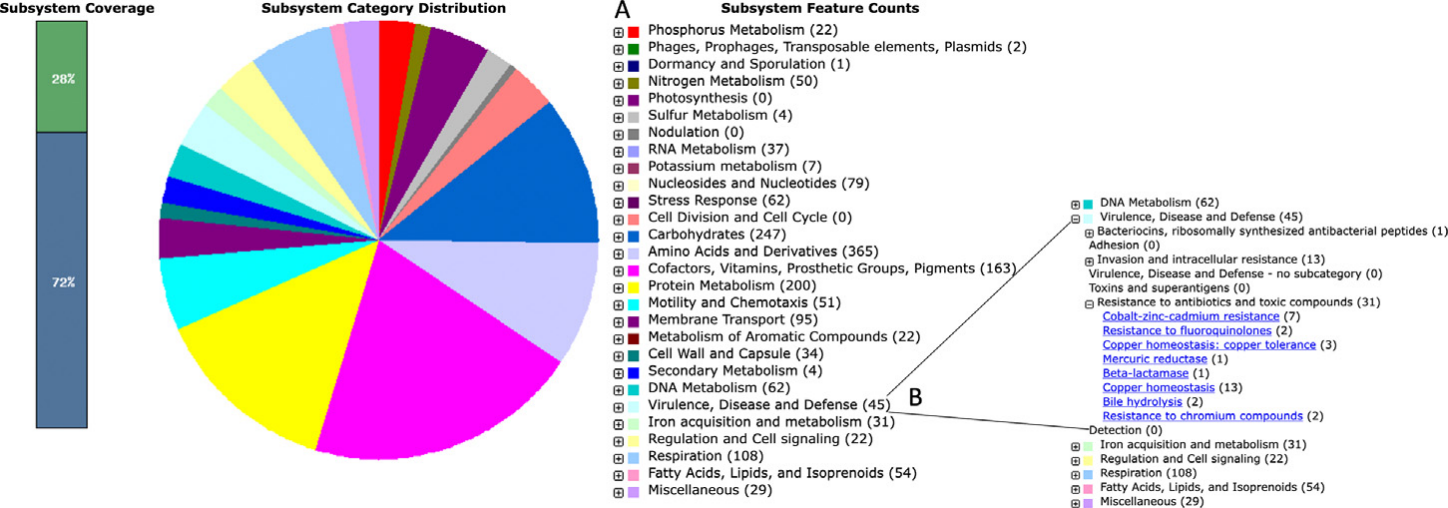
Summary of the subsystem categories of *Ochrobactrum anthropi strain* Nas42, obtained using the RAST annotation web server. (A) 25 functional groups were identified and classified with RAST annotation. (B) Gene group associated with metal tolerance. The subsystem categories and corresponding counts are presented with distinct colors.

To obtain a subsystem-level overview, the genome of *O. anthropi* strain Nas42 was further annotated using the RAST server. RAST assigned approximately 28 % of the genes to 25 distinct functional subsystems (Fig. 4), providing a structured view of key metabolic and stress-response capabilities in this strain. Among these 25 subsystems, the “amino acids and derivatives” category contained the highest number of genes (365), followed by “carbohydrate metabolism” (247 genes), “protein metabolism” (200 genes), and “cofactors, vitamins, prosthetic groups, and pigments” (163 genes). In contrast, categories such as potassium metabolism (7 genes), secondary metabolism (4 genes), sulfur metabolism (4 genes), and dormancy and sporulation (1 gene) were represented by relatively few genes.

Several subsystems related to elemental cycling were identified, including genes involved in nitrogen metabolism (32 genes), phosphorus metabolism (22 genes), and sulfur metabolism (5 genes).

Furthermore, RAST annotation identified 45 genes within the “virulence, disease and defense” subsystem that are associated with toxic metals. These include 3 genes related to cobalt–zinc–cadmium resistance, 16 genes involved in copper homeostasis and resistance, 2 genes linked to resistance to chromium compounds, and 1 gene encoding a mercury reductase.

## 4 Experimental Design, Materials and Methods

### 4.1 Isolation and culture of strain Nas42

Strain Nas42 was isolated from estuarine sediment samples collected on 29 May 2024 at station N24 (20°13.000′ N, 106°37.520′ E), located at the mouth of the main branch of the Red River in the Ba Lat estuary, in the Red River Delta, northern Vietnam during the PLUME oceanographic campaign (May-July 2024) [8]. Selective cultures were performed from 5 mL sediments in 45 mL medium containing (per liter): 0.3 g KH₂PO₄, 0.3 g K₂HPO₄, 0.5 g NH₄Cl, 6 g NaCl, 1 g MgSO₄, 1 g NaNO₃, 0.1 g KCl, 0.1 g CaCl₂·2H₂O, 0.5 mM glucose, 0.5 mM pyruvate, 0.5 mM fumarate, 0.5 mM lactate, 0.5 mM ethanol, 0.1 % (v/v) trace elements (SL-10), and 1 % (v/v) Balch’s vitamins [9]. Selection for metal tolerance was achieved by supplementing with 5 mM CdCl₂ and 0.1 mM arsenic. Cultures were maintained under microaerobic conditions at 30 °C without shaking. The pH was adjusted to 6.5. After three subcultures in the selective medium, isolation was performed using the Hungate technique with roll-tube cultivation in the same medium with agar added at 1.5 % (w/v), in microaerobic conditions [10]. A colony (Nas42) was selected and cultivated in 5 mL liquid medium for 72 h at 30 °C, followed by 10^−2^ serial dilutions until extinction.

### 4.2 Genomic DNA extraction and quality assessment

For genome sequencing, DNA was extracted using the Wizard Genomic DNA Purification Kit (Promega), following manufacturer’s recommendations, from 5 mL of culture in medium without the addition of metals. DNA quality was assessed using NanoDrop spectrophotometry (A₂₆₀/A₂₈₀ ratio: 1.8–2.0; A₂₆₀/A₂₃₀ ratio: >2.0) and 1 % (w/v) agarose gel electrophoresis.

### 4.3 Whole-Genome sequencing and assembly

Whole-genome sequencing was performed on the Illumina MiSeq platform at KTEST SCIENCE CO. LTD (Ho Chi Minh City, Vietnam) using MiSeq Reagent Kit v3 with 150 bp paired-end chemistry. Raw read quality was initially assessed with FastQC (http://www.bioinformatics.babraham.ac.uk/projects/fastqc/) to examine per-base quality scores, GC content, and adapter contamination. Quality trimming and filtering were performed with Trimmomatic v0.39 [11] using a sliding-window approach, removing bases with Phred quality scores below Q30 and discarding reads shorter than 50 bp. *De novo* assembly was performed using SPAdes v3.2.0 (https://github.com/ablab/spades/releases, accessed in November 2025). Assembled sequences were then validated using Unicycler v0.5.1 (https://github.com/rrwick/Unicycler/releases, accessed in November 2025).

### 4.4 Genome quality assessment and annotation

Genome completeness and quality were evaluated using QUAST v5.3.0 [12] and BUSCO v5.8.0 [13]. Contamination levels were assessed using CheckM2 v1.0.2 [14]. Repeat sequences were identified using RepeatMasker v4.1.7 (https://github.com/Dfam-consortium/RepeatMasker, accessed in November 2025). Protein-coding sequences were predicted using Prokka v1.14.6 with bacterial genetic code 11 [15]. All bioinformatics tools and software were run using their default parameters unless otherwise specified. Circular genome visualization was performed using CGView server (https://proksee.ca/, accessed in November 2025). Annotation and gene prediction from the assembled genome were performed using the Rapid Annotations using Subsystems Technology (RAST) [16].

### 4.5 Functional annotation

Predicted protein-coding sequences from the Nas42 genome were functionally annotated by similarity search against three reference databases: Clusters of Orthologous Genes (COG, http://eggnog-mapper.embl.de/), Kyoto Encyclopedia of Genes and Genomes (KEGG, http://www.kegg.jp/), and Gene Ontology (GO, http://www.geneontology.org/). Protein sequences were aligned to these databases using DIAMOND in BLASTP mode with an E-value cutoff of ≤ 1 × 10⁻⁵, and the best-scoring hits were retained for downstream annotation. Based on the presence or absence of valid hits in each database, a binary annotation matrix (COG/KEGG/GO) was generated for all CDSs, and the overlap among the three datasets was summarized and visualized as a Venn diagram using the EVenn web tool (https://www.bic.ac.cn/EVenn/).

### 4.6 Taxonomic classification

Whole-genome-based taxonomic analysis was then conducted with closely related type strains using the Type Strain Genome Server (TYGS; https://tygs.dsmz.de/) [17], which computes intergenomic distances and reconstructs a phylogenomic tree based on the Genome BLAST Distance Phylogeny (GBDP) method. The resulting phylogenomic tree was visualized using the iTOL web server (https://itol.embl.de/, accessed in November 2025), allowing the position of Nas42 to be examined relative to reference *Brucella* and *Ochrobactrum* genomes.

Average Nucleotide Identity (ANI) values between Nas42 and selected reference genomes were calculated using the ANIb algorithm implemented on the jSpeciesWS web server (https://jspecies.ribohost.com/jspeciesws/#analyse, accessed in November 2025) to quantify genome-wide similarity and assess species-level relatedness. In parallel, the presence or absence of the insertion sequence IS711, a hallmark of true *Brucella* species, was evaluated by BLAST searches against the Nas42 genome and representative *Brucella* and *Ochrobactrum* genomes. The combination of phylogenomic placement (TYGS/GBDP), ANIb values, genome size comparisons, and IS711 screening was used to distinguish true *Brucella species* from *Ochrobactrum* lineages.

## Limitations

The dataset is based on the genome sequencing of a single isolate. A single genome cannot capture the genetic diversity within a species (the pan-genome), phylogenetic relationships, or the evolutionary dynamics (e.g., clonal expansion vs. horizontal gene transfer) within a species. For a more robust dataset, multiple isolates (not clones) belonging to the same species should be subjected to genome sequencing for greater insight into the genetic potential of the pan-genome.

## Ethics Statement

The authors have read and follow the ethical requirements for publication in Data in Brief and confirm that the current work does not involve human subjects, animal experiments, or any data collected from social media platforms.

## CRediT Author Statement

**Duong Huy Nguyen**: Methodology, Software, Formal analysis, Data Curation, Writing - Original Draft, Writing - Review & Editing; **Nathalie Pradel**: Methodology, Investigation, Formal analysis, Data curation, Validation, Writing - Original Draft, Writing - Review & Editing; **Sandrine Chifflet**: Methodology, Sampling, Writing - Review & Editing; **Marc Tedetti:** Methodology, Sampling**,** Writing - Review & Editing, funding; **Ngoc Bich Pham**: Methodology, Supervision, Writing - Review & Editing; **Van Ngoc Bui**: Methodology, Investigation, Data Curation, Supervision, Validation, Writing - Review & Editing.

## Data Availability

Genebank, SRAWhole genome sequence of Ochrobactrum anthropi strain Nas42 from estuarine sediments of the Red River Delta, Vietnam (Original data)
